# Prenatal exposure to bisphenol A impacts neuronal morphology in the hippocampal CA1 region in developing and aged mice

**DOI:** 10.1007/s00204-015-1485-x

**Published:** 2015-03-25

**Authors:** Eiki Kimura, Chieri Matsuyoshi, Wataru Miyazaki, Seico Benner, Mayuko Hosokawa, Kazuhito Yokoyama, Masaki Kakeyama, Chiharu Tohyama

**Affiliations:** 1 Laboratory of Environmental Health Sciences, Center for Disease Biology and Integrative Medicine, Graduate School of Medicine, The University of Tokyo, Tokyo, Japan; 2 Department of Epidemiology and Environmental Health, Juntendo University Faculty of Medicine, Tokyo, Japan; 3 Graduate School of Biomedical Science, Nagasaki University, Nagasaki, Japan

**Keywords:** Bisphenol A, Dendrite, Developmental neurotoxicity, Hippocampus, Spine

## Abstract

Bisphenol A (BPA), a widely used raw component of polycarbonate plastics and epoxy resins, has been reported to induce developmental neurotoxicity in offspring born to dams exposed to low doses of BPA; however, the toxicity mechanism remains elusive. To study the effects of in utero BPA exposure on neuronal morphology, we studied spine density and dendritic growth in the hippocampal CA1 of aged mice and developing mice prenatally exposed to low doses of BPA. Pregnant mice were orally administered BPA at a low dose of 0, 40, or 400 μg/kg body weight/day on gestational days 8.5–17.5/18.5. Mouse progenies were euthanized at 3 weeks or 14 months, and their brains were analyzed for dendritic arborization of GFP-expressing neurons or spine densities of Golgi-stained neurons in the hippocampal CA1. Regardless of the dose, in utero BPA exposure reduced spine densities in the hippocampal CA1 of the 14-month-old mice. In the developing brain from the 3-week-old mice born to dams exposed to BPA at a dose of 400 μg/kg body weight/day, overall length and branching number of basal dendrites but not apical dendrites were decreased. In utero low doses of BPA exposure disrupts hippocampal CA1 neuronal morphology during development, and this disruption is believed to persist in adulthood.

**Electronic supplementary material:**

The online version of this article (doi:10.1007/s00204-015-1485-x) contains supplementary material, which is available to authorized users.

## Introduction

The occurrence of neurodevelopmental disabilities, including autism spectrum disorder (ASD), attention-deficit/hyperactivity disorder, dyslexia, and other cognitive impairments, has been reported to be increased in several countries (Fombonne 2009; Grandjean and Landrigan 2014; Newschaffer et al. 2007; Polanczyk et al. 2007; Tchaconas and Adesman 2013). The estimated prevalence of ASD, a serious and common neurodevelopmental disorder, is 4–5 per 10,000 births (Fombonne 1999), with a drastic increase at least 1–2 per 1,000 births (Chakrabarti and Fombonne 2005; Yeargin-Allsopp et al. 2003). A more recent report estimated an incidence of 14.7 per 1,000 children aged 8 years in 2010, although the lack of a standardized widely accepted indicator of severity was noted (Developmental Disabilities Monitoring Network Surveillance Year Principal I, Centers for Disease C, Prevention 2014). Although the real cause of this increase in ASD incidence remains largely unknown, intrinsic factors such as genetic disposition and social factors such as amended diagnostic criteria cannot solely account for such a drastic increase in recent years (Lawler et al. 2004; McDonald and Paul 2010). Recently, the impact of a stressful environment during fetal and infantile periods on health and disease states later in life has been recognized under the theory of “developmental origins of health and disease (DOHaD)” or the concept of “fetal origins of adult disease (FOAD)” (Calkins and Devaskar 2011; Wadhwa et al. 2009). A large body of evidence generated through epidemiological and clinical studies has shown that environmental chemical exposure at least partly contributes to the etiology of neurodevelopmental disorders and related mental problems (Grandjean and Landrigan 2014).

Brain development includes both dendritic growth and generation of neuronal connections to form a neural network (McAllister et al. 1997; Rihn and Claiborne 1990; Wong and Ghosh 2002). These developmental processes occur within a finely controlled time frame, in which all developmental stages are orchestrated to proceed on schedule and in the correct sequence (Rice and Barone Jr. 2000). Each brain region is responsible for various functions, and histological injury to any brain region impairs specific functions (Fujimoto et al. 2004; McAllister et al. 2004). The hippocampus consists of four main histological subregions, CA1, CA2, CA3, and dentate gyrus (DG), and it has been extensively studied as a part of the brain system responsible for learning and memory (Bannerman et al. 2014; Moser et al. 2014). Pyramidal neurons in CA1 and CA3 consist of basal and apical dendrites that differ morphologically and biophysically (Bannister and Larkman 1995; Magarinos et al. 1996) and play an important role in neural circuitry, thus promoting hippocampal function (Nolan et al. 2004). Axonal projections of the CA3 pyramidal neuron, also known as Schaffer collateral fibers, form connections on the dendritic spines of CA1 pyramidal neurons (Amaral and Witter 1989). Dendritic spines are small protrusions extending from the neuronal dendrites and bear the majority of excitatory synapses, where glutamate receptors, including *N*-methyl-d-aspartate (NMDA) and α-amino-3-hydroxy-5-methyl-4-isoxazolepropionic acid (AMPA) receptors, are cardinal components of postsynaptic specialization (Petralia and Wenthold 1992; Petralia et al. 1994). The aged mice exhibit marked reductions in the numbers of basal CA1 dendrite spines and length of apical CA1 dendrite spines and DG neurons, all of which are associated with memory and cognitive impairment (von Bohlen und Halbach et al. 2006).

Bisphenol A (BPA) is a raw component of the polycarbonate plastics and epoxy resins that are used to manufacture plastic containers such as baby bottles (Brede et al. 2003) and food and drink can linings (Kang et al. 2003). Because of a large volume of production of BPA, it is widespread in the environment and was reported to be detected in biological specimens including amniotic fluid, suggesting that humans can be exposed to low levels of BPA from fetal to adult stages (Ikezuki et al. 2002; vom Saal and Hughes 2005; Vandenberg et al. 2012). BPA binds classical nuclear, as well as membrane-associated estrogen receptors (ERs), and may induce adverse health effects by disrupting ER signaling (Wetherill et al. 2007).

Large-scale two-generation reproduction studies conducted according to the so-called good laboratory practices have demonstrated that perinatal exposure to BPA doses as low as 50 mg/kg body weight (b.w.)/day did not induce any adverse effects (Ema et al. 2001; Tyl et al. 2002); accordingly, this dose was designated as the no observed adverse effect level (NOAEL) and was used to derive a tolerable daily intake (TDI) value of 50 μg/kg b.w./day. In contrast, small-scale studies conducted primarily in university laboratories revealed that doses similar to or lower than the NOAEL or TDI could induce reproductive, immunological, and central nervous system abnormalities in rats and mice (Melnick et al. 2002). Regarding the central nervous system, the mechanism by which BPA-induced altered behavior correlates with morphology remains unknown.

In in vivo animal models of BPA-induced developmental neurotoxicity (DNT), perinatal exposure to a low-dose BPA impairs higher brain function. BPA-exposed rats and mice were found to exhibit persistent learning and memory impairments (Poimenova et al. 2010; Tian et al. 2010; Xu et al. 2012), anxiety-like behavior (Luo et al. 2014), and social recognition difficulties (Wolstenholme et al. 2013). In these BPA-exposed rodent models, hippocampal expression of the AMPA (subunit GluR1) and NMDA receptors was downregulated (Tian et al. 2010; Xu et al. 2012). Neither the DOHaD nor FOAD theory has been challenged by whether the perinatal BPA exposure-induced hippocampal impairment observed in young animals would persist through aging. Here, we report that in utero exposure to a low-dose BPA impaired not only the dendritic arborization of CA1 pyramidal neurons at an earlier developmental stage but also dendritic spine density in the hippocampal CA1 region during adulthood.

## Materials and methods

### Reagents and chemicals

BPA (purity ≥99 %) was purchased from Sigma-Aldrich Chemical Co (St Louis, MO, USA). Corn oil was purchased from Wako Pure Chemical (Osaka, Japan). The manufacturers of other reagents and instruments used in this study are described in each section below.

### Animals and treatment

C57BL/6J mice were purchased from CLEA Japan (Tokyo, Japan); mice from the Thy1-GFP-M line (Thy1-GFP-M mice, hereafter) were a kind gift from Dr. G. Feng at Massachusetts Institute of Technology (Feng et al. 2000). Female C57BL/6J wild-type mice were mated with male Thy1-GFP-M mice to produce pups bearing Thy1-GFP allele. These mice were housed in an animal facility with a set temperature of 22–24 °C and humidity of 40–60 % as well as a 12-/12-h light/dark cycle (lights on from 08:00 to 20:00). Laboratory rodent chow (Lab MR Stock, Nosan, Yokohama, Japan) and distilled water were provided ad libitum.

Pregnant female wild-type mice were orally administered vehicle (corn oil) or BPA in vehicle at a dose of 0, 40, or 400 μg/kg b.w./day from gestation day (GD) 8.5–18.5 (doses correspond to groups named hereafter as control, BPA-40, and BPA-400, respectively). The pups were culled to 6–8 per dam on postnatal day (PND) 0, weaned on PND 21, and maintained under the same conditions as their dams. One male pup per dam was randomly selected for the analysis of dendritic morphology to minimize possible litter effects (*n* = 4–5 mice/group). The animal experimental protocols used in this study were approved by the Animal Care and Use Committee of the University of Tokyo.

### Golgi staining of neurons in aged mice

**Fig. 1:**
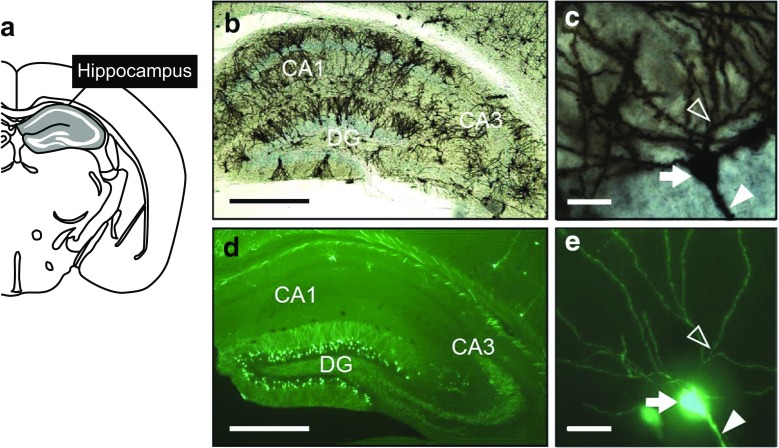
**a** Regions of interest. This illustration has been modified with permission from “The mouse brain in stereotaxic coordinates” (Paxinos and Franklin 2004). **b**, **c** Representative photomicrographs of the CA1 region of the hippocampus (**b**) and a CA1 pyramidal neuron in a Golgi-stained brain. **d**, **e** Representative photographs of the CA1 region of the hippocampus (**d**) and CA1 pyramidal neurons in a Thy1-GFP-M mouse. *Arrows* indicate cell bodies, *open arrowheads* indicate basal dendrites, and *filled arrowheads* indicate apical dendrites. *Bar* 500 μm in **b** and **d**, 20 μm in **c** and **e**

Golgi staining of neurons was performed using the FD Rapid Golgi Stain Kit (FD NeuroTechnologies, Ellicott City, MD, USA) according to the manufacturer’s instructions. Briefly, male mice (14 months old) were killed by cervical dislocation. Brains were quickly removed from the skulls, briefly rinsed in distilled water, and incubated in a mixture of A and B solutions from the kit for 3 days at room temperature in the dark, after which they were transferred into solution C from the kit for 3 days at 4 °C. The brain tissues were sectioned through the coronal plane of the hippocampus into 150-μm-thick slices using a cryostat (Leica CM3050S, Leica Microsystems K. K., Tokyo, Japan). Brain sections were placed on gelatinized slides, rinsed in distilled water, and immersed in a mixture of D and E solutions from the kit and distilled water for staining in the dark, followed by another rinse in distilled water. The slides were subsequently dehydrated in a series of ethanol solutions (50, 70, 90, 100 %) for 5 min each, covered with Permount mounting media (Fisher Scientific, Pittsburgh, PA, USA), and stored in the dark until completely dry. The hippocampus of the brain sections were finally viewed under a microscope (Leica DM6000 B, Leica Microsystems, Wetzler, Germany; Fig. 1a–c).

### Preparation of brain tissues from Thy1-GFP-M mice

On PND 21, male Thy1-GFP-M mice were anesthetized with sodium pentobarbital and transcardially perfused with 4 % paraformaldehyde (PFA) in 0.1 M phosphate-buffered saline (PBS, pH 7.4). The brains were removed and fixed overnight in 4 % PFA. Routinely, brains were immersed in a series of 5, 15, and 30 % sucrose in 0.1 M PBS, frozen in Tissue-Tek OCT compound (Sakura Finetek, Tokyo, Japan), and stored at −80 °C until analysis. Brains were sectioned through the coronal plane of the hippocampus into 300-μm-thick slices with a sliding microtome (Yamato Koki, Tokyo, Japan). Brain sections were collected and rinsed in 0.1 M PBS and placed on slides. Each slide was covered with Vectashield (Vector Laboratories, Burlingame, CA, USA) and a plastic coverslip and viewed with a Leica DM6000 B microscope (Fig. 1d, e).

### Analysis of dendritic morphology

The pyramidal neuronal structures in the hippocampal CA1 regions of Golgi-stained brain tissues and GFP-Thy1-M brains were subjected to a morphological analysis using Neurolucida software, a computer-based neuron tracing system (MicroBrightField, Colchester, VT, USA). A single neuron was traced under a Leica DM6000 B microscope with an objective lens (HCX PL APO, 40×, NA = 0.75; Leica Microsystems). GFP-expressing neurons were observed via camera lucida at 400-fold magnification under blinded conditions (i.e., information about the tissue sections was kept confidential from that analyzing cell morphology). The three-dimensional morphologies of basal and apical dendrites were also quantified using Neurolucida software. The 1st–5th dendritic branches were named according to the proximity to the cell body. A three-dimensional Sholl analysis was used to reveal the dendritic branching pattern complexity and to assess differences in dendrite locations by counting the numbers of intersections between dendrites and an overlaid concentric sphere at 10-μm intervals. Using NeuroExplorer software (MicroBrightField), the total lengths, numbers of branches, and branching patterns of basal and apical dendrites were quantified and compared across groups. Five to eight neurons were traced per mouse; the total numbers of neurons analyzed for dendritic arborization were 31, 29, and 31 in the control, BPA-40, and BPA-400 groups, respectively. The CA1 region was subjected to dendritic spine density analysis under 400-fold magnification. All visible spines along the selected dendritic segments were counted, and spine density values were expressed as the number of spines per 10-μm dendrite. Five to 22 segments were counted per mouse; the total numbers of segments subjected to spine density analysis were 76, 79, and 75 in the control, BPA-40, and BPA-400 groups, respectively.

### Statistical analysis

All statistical analyses were conducted with an SPSS 15.0 (SPSS Japan Inc, Tokyo, Japan). The entire dendrite lengths, numbers of branches, and spine densities were analyzed using a one-way analysis of variance (ANOVA), followed by the Tukey–Kramer post hoc test. A two-way repeated-measures ANOVA was used to compare Sholl analyses between groups. *P* values of <0.05 were considered statistically significant.

## Results

### Spine densities of dendrites in the brains from aged offspring of BPA-exposed mice

**Fig. 2:**
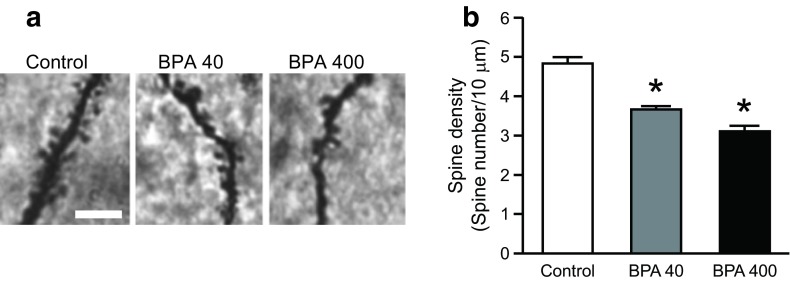
Spine densities of dendrites in the hippocampal CA1 of 14-month-old mice. **a** Representative photomicrographs showing Golgi-stained dendrites and spines in the CA1. *Bar* 10 μm. **b** Decreased spine density [i.e., the number of spines per arbitrary dendrite length (10 μm)] in the brains of mice from the BPA-40 and BPA-400 groups. *Asterisks* indicate significant differences from the control group (*p* < 0.05). Values are shown as the means ± standard errors of the mean (SEM) for four mice per group

In utero BPA exposure yielded a remarkable decrease in the spine density of the hippocampal CA1 of 14-month-old male offspring (Fig. 2a). Morphometric analysis revealed significant reductions in spine density in both the BPA-40 and BPA-400 groups compared with that in the control group [one-way ANOVA, *F*(2, 9) = 36.5, *p* < 0.0001; with a Tukey–Kramer post hoc test, *p* < 0.05; Fig. 2b]. There was no significant difference in spine density between the BPA-40 and BPA-400 groups.

### Dendrite arborization in the brains from developing offspring of BPA-exposed mice

**Fig. 3:**
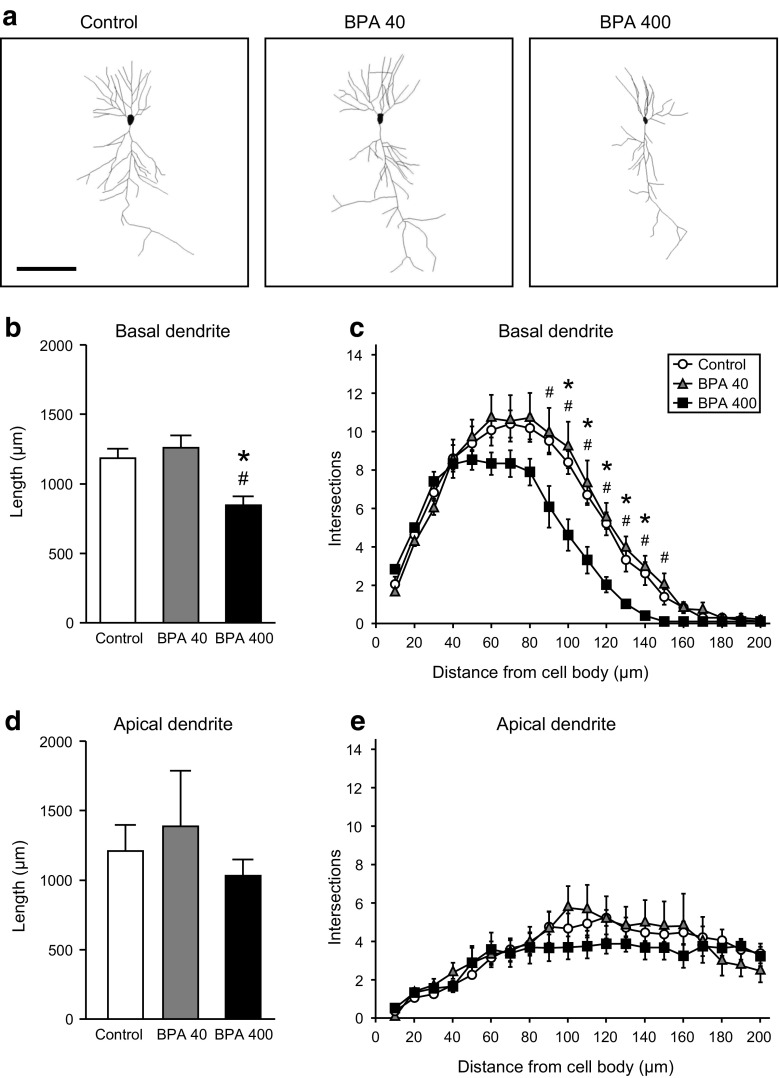
Entire lengths and branching patterns of basal and apical dendrites from the hippocampal CA1 pyramidal neurons of 21-day-old GFP-Thy1-M mice prenatally exposed to BPA. GFP-positive CA1 pyramidal neurons were traced using Neurolucida software. **a** Images of the camera lucida drawings of CA1 pyramidal neurons from the control, BPA-40, and BPA-400 groups. *Bar* 100 μm. **b–e** The entire lengths (**b**, **d**) and branching patterns (**c**, **e**) of basal (**b**, **c**) and apical (**d**, **e**) dendrites. Symbols (* and ^#^) indicate significant differences between the BPA-400 group and the control and BPA-40 groups, respectively (*p* < 0.05). Values are shown as mean ± SEM for five mice per group

Neurolucida software was used to trace the contours of basal and apical dendrites on GFP-positive CA1 pyramidal neurons from Thy1-GFP-M mice born to dams administered corn oil or BPA (Fig. 3a). Basal and apical dendrite morphologies were analyzed according to the following parameters: (1) the entire length of each dendrite, (2) branching pattern complexity, and (3) number of branches in a specified order on each dendrite.

In utero BPA exposure significantly affected the entire basal dendrite lengths on hippocampal CA1 pyramidal neurons from 21-day-old mice [one-way ANOVA, *F*(2, 12) = 7.55, *p* < 0.01]. The entire basal dendrite lengths in the BPA-400 group were 28 and 33 % shorter than those in the control and BPA-40 groups, respectively (Fig. 3b; Tukey–Kramer post hoc test, *p* < 0.05). The basal dendrite branching pattern complexity was determined as described above; this complexity was significantly impaired by in utero BPA exposure (Fig. 3c). Two-way repeated-measures ANOVA revealed significant differences for both BPA exposure and distance from the cell body [*F*(2, 297) = 39.2, *p* < 0.0001 and *F*(19, 280) = 111.7, *p* < 0.0001, respectively] and a significant interaction between the two main factors [*F*(38, 261) = 2.45, *p* < 0.0001]. The numbers of intersections between basal dendrites and concentric circles at 10-μm intervals 90–150 μm from the cell body were compared across groups. In the BPA-400 group, the numbers of intersections were significantly decreased at each concentric circle between 100 and 140 μm from the cell body (Tukey–Kramer post hoc test, *p* < 0.05) relative to those in the control group and between 90 and 150 μm from the cell body relative to those in the BPA-40 group (Tukey–Kramer post hoc test, *p* < 0.05). No significant differences in the entire length and complexity were observed between the control and BPA-40 groups. In contrast to basal dendrites, apical dendrites were not significantly affected by in utero BPA exposure in terms of the entire length and complexity when compared to the control group (Fig. 3d, e).

**Fig. 4:**
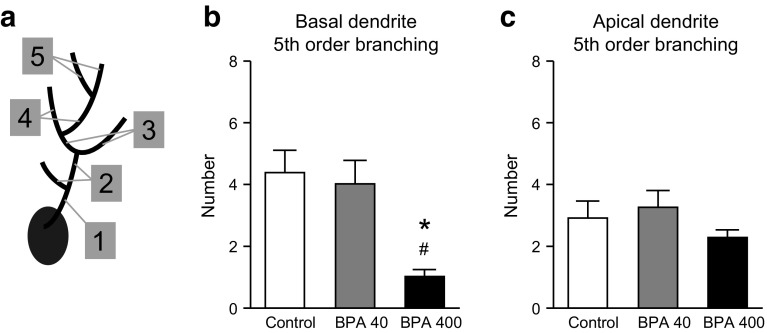
Number of 5th-order branches of basal and apical dendrites on hippocampal CA1 pyramidal neurons from 21-day-old GFP-Thy1-M mice prenatally exposed to BPA. **a** Scheme for assigning branch order to a dendritic tree. The segment beginning at the dendrite origin is designated 1st-order branching. **b**, **c** Numbers of 5th-order branches from basal dendrites (**b**) and apical dendrites (**c**) of CA1 pyramidal neurons. Symbols (* and ^#^) indicate significant differences between the BPA-400 group and the control and BPA-40 groups, respectively (*p* < 0.05). Values are shown as mean ± SEM for five mice per group

Next, basal and apical dendrites were analyzed with respect to the number of branches in 1st–5th order (Fig. 4a). No differences in the numbers of branches were observed among 1st–4th basal dendrites (Fig. S1a–d). However, a one-way ANOVA revealed a significant reduction in the number of 5th-order branches in the BPA-400 group relative to the control and BPA-40 groups [*F*(2, 12) = 7.78, *p* < 0.01; Tukey–Kramer post hoc test, *p* < 0.05; Fig. 4b]. No alterations were observed between the control and BPA groups with respect to the numbers of 1st- to 4th-order apical dendrite branches (Fig. S1e–h) or in 5th-order branches (Fig. 4c).

## Discussion

Epidemiological and clinical studies have generated a large body of evidence, demonstrating that environmental chemical exposure has at least partly contributed to the etiology of neurodevelopmental disabilities. Although a few industrial chemicals, such as methylmercury, lead, and polychlorinated biphenyls, have been established as the causes of neurodevelopmental disabilities and subclinical brain dysfunction, these represent the tip of the iceberg of potential chemical hazards that might disrupt the developing brain (Grandjean and Landrigan 2006). Exposure to these chemicals during early fetal development can cause brain injuries at doses much lower than those that affect adult brain function. In the present study, prenatal exposure to low doses of BPA was observed to impair the brain structure in terms of dendritic spine density, total dendritic length, and dendritic branching numbers not only in infant mice but also in aged mice, indicating that morphological alterations that occurred at an early life stage remained until later in life. As the estimated elimination half-life of BPA in the mouse is approximately 6 h (Sieli et al. 2011), it is likely that such abnormalities are initiated during gestation rather than later in life. Our findings might support the theories of DOHaD or FOAD (Calkins and Devaskar 2011; Wadhwa et al. 2009), in which challenges posed by environmental stresses during gestation might facilitate the onset of a disease state in adulthood.

Dendritic spines bear the majority of excitatory synapses wherein glutamate receptors, such as the NMDA and AMPA receptors, are cardinal components of postsynaptic specialization (Petralia and Wenthold 1992; Petralia et al. 1994). Schaffer collateral fibers, which are primary projections from the CA3 pyramidal neurons to the dendritic spines of CA1 pyramidal neurons, represent the critical circuit of effective glutamatergic synaptic transmission (Amaral and Witter 1989). The decreased hippocampus CA1 dendritic spine density observed in the aged mice exposed to BPA in utero (Fig. 2a, b) is believed to reflect synaptic dysfunction between the CA1 and CA3 neurons. A few reports have indicated that developmental exposure to BPA affects the hippocampal glutamatergic system and hippocampal function. For example, binding to a number of NMDA receptors in hippocampal CA1, CA3, and DG was observed to be reduced in mice orally administered BPA at daily doses of 100 or 500 μg/kg b.w. from PND 7 to 36 (Tian et al. 2010); however, the results of that study should be interpreted cautiously because only two dams were used per group. The expression of the hippocampal AMPA receptor GluR1 subunit was found to be decreased in mice that were administered BPA at a daily oral dose of 0.4 and 4.0 mg/kg b.w. either from GD 7 to GD 20 or from PND 1 to PND 14 (Xu et al. 2012). Furthermore, mice administered BPA at a daily oral dose of 100 or 500 μg/kg b.w. and rats administered BPA at a daily oral dose of 40 μg/kg b.w. were observed to exhibit impaired working memory and spatial recognition memory, respectively (Poimenova et al. 2010; Tian et al. 2010), although the underlying mechanisms remain elusive. Our present study provides histological evidence in support of the notion that perinatal BPA exposure reduces the expression of glutamate receptors, induces abnormal processing between neurons, and impairs higher brain function.

A decrease in spine density may elicit weaker postsynaptic responses to a given stimulus, resulting in deficits in higher brain function (Garey et al. 1998; Perez-Cruz et al. 2011). Because neuronal activity in the hippocampal CA1 region is activated through spatial recognition and novel environmental stimuli, as shown using laser microdissection and quantitative RT-PCR analysis (Yoshioka et al. 2012), morphological abnormalities in neuronal dendrites of the CA1 region could possibly reduce hippocampal activity. In fetal monkeys, prenatal BPA exposure reduced the number of dopaminergic neurons in the midbrain and several spine synapses in the hippocampal CA1 region; however, BPA administration to juvenile monkeys did not elicit any effects on these indices (Elsworth et al. 2013). In contrast, our present study showed significant decreases in spine density in aged BPA-exposed mice, implying that the effect of fetal BPA exposure remains as an epigenetic memory later in adulthood. Aging alone is accompanied by a significant loss in basal CA1 dendrite spine numbers, a phenomenon that has been associated with a decline in hippocampal function in C57BL/6 mice (von Bohlen und Halbach et al. 2006). Our findings indicated that the decrease in spine density in adulthood could be attributed not only to aging but also to in utero BPA exposure. In previous in vitro studies of cultured hippocampal slices, however, BPA exposure was observed to rapidly affect pyramidal neurons through a mechanism mediated by ER activity (Tanabe et al. 2012; Xu et al. 2014). Therefore, the in vivo data reported thus far, including our present results, appear consistent in terms of the morphological impairments induced by prenatal BPA exposure and the association of such exposure with accelerated age-related impairments in brain structure and function.

During brain development, neurons must migrate in a precise manner from their points of origin to their assigned destinations and therein extend dendrites and axons to establish connections with other neurons via synapses (McAllister et al. 1997; Rihn and Claiborne 1990; Strittmatter et al. 1995; Wong and Ghosh 2002). Normal dendritic growth is an essential component of neural network formation, and perturbed dendritic growth induces abnormal higher brain function in laboratory animal models of neurodevelopmental disabilities such as ASD and schizophrenia (Broadbelt et al. 2002; Garey et al. 1998; Harrison 2004; Hutsler and Zhang 2010). In the present study, decreases in the entire length and branch number of basal dendrites were observed on CA1 pyramidal neurons from the developing brains of the BPA-400 group when compared to dendrites from the control and BPA-40 groups (Figs. 3, 4), suggesting insufficient synapse formation in the hippocampal region resulting from increased BPA exposure and consequent decreased dendrite surface area and implying a deficit in the hippocampal circuit related to memory and recognition.

In neurons, dendritic growth is regulated by noradrenaline, which is released from the axon terminals of neurons in the locus ceruleus (LC) (Kolb et al. 1997). For example, when rats subjected to LC removal on PND 0 were compared with sham-operated rats, the percentage of ascending pyramidal neurons was observed to markedly increase in the former group along with a marked decrease in the branching number (Maeda et al. 1974). Kubo et al. (2003) showed that exposure to 0.1 or 1.0 ppm BPA (equal to a daily oral dose of 30 or 300 μg/kg b.w., respectively) impaired the LC size and thus might have perturbed the growth of noradrenaline axons originating from the LC. Because noradrenergic neurons project throughout the telencephalon, including the cerebral cortex and hippocampus, dendritic growth in the BPA-400 group (Figs. 3, 4) might have been retarded by the disruption of the noradrenaline-releasing system in the LC.

Neuronal development is significantly affected by neurosteroids such as estradiol, suggesting that ER signaling is crucial for neural network formation (Sakamoto et al. 2003; Sasahara et al. 2007). BPA has been known to interact with ERα and ERβ, and has been shown to interfere with hippocampal synaptogenesis (Leranth et al. 2008; MacLusky et al. 2005). Although the affinity of BPA with ERs seems to be low as shown in in vitro assay (Kuiper et al. 1998), recent studies revealed that BPA can modulate spinogenesis and synaptic plasticity via estrogen-related receptor gamma (ERRγ), not by estrogen receptors (ERα/ERβ) in hippocampal neuron slice experiments (Hasegawa et al. 2013; Tanabe et al. 2012). Because of a short elimination half-life of BPA, it is not likely that BPA administered in utero remained during the infantile period or later, and thus, it is speculated that the abnormal dendritic spine morphology observed in infantile mice as well as aged mice may have originated by the disrupted ER signaling during gestation.

To assess the DNTs of chemicals, endpoints described in terms of structural alterations can provide additional information regarding prenatal and/or postnatal central nervous system damage. Neuronal morphology can be used to evaluate a wide range of temporal effects of the DNT. Present observations regarding morphological alterations in both developing mice and aged mice exposed to BPA in utero suggest that the altered brain morphology induced in utero persists in adulthood. A quantitative neuromorphological analysis of the effects of perinatal chemical exposure reveals relationships between the structural disruption and functional impairments in the brain, allowing a better understanding of the DNT.

In this study, we have shown that in utero BPA exposure alters the dendritic arborization of CA1 pyramidal neurons in the developing brain and that spine density in the hippocampal CA1 is decreased in the brains of aged mice subjected to prenatal BPA exposure. DNT guidelines published by the Organisation for Economic Cooperation and Development (OECD) and the U.S. Environmental Protection Agency (EPA) indicate that neuropathological examinations can be conducted using hematoxylin and eosin or silver staining but do not mention morphological analysis at a single-neuron level (OECD 2007; US EPA 1998). Our results suggest that neuronal morphology is an important endpoint of DNT studies. In addition, morphological analysis will help to confirm behavioral and molecular alterations resulting from environmental chemical exposure and facilitate an understanding of neurodevelopmental disorders and related health problems in terms of the DOHaD and FOAD viewpoints.

## Electronic supplementary material

Below is the link to the electronic supplementary material.

Supplementary material 1 (DOCX 233 kb)
