# Supplementary material for: Prenatal exposure to bisphenol A impacts neuronal morphology in the hippocampal CA1 region in developing and aged mice
Source: Arch Toxicol. 2015 Mar 25;90(3):691–700. doi: 10.1007/s00204-015-1485-x (PMC4754327; doi:10.1007/s00204-015-1485-x)
Supplement: Supplementary file 1 — Supplementary material 1 (DOCX 233 kb) [file 204_2015_1485_MOESM1_ESM.docx]

**Supplementary Material**

**Article title:** Prenatal exposure to bisphenol A impacts neuronal morphology in the hippocampal CA1 region in developing and aged mice

**Journal name:** Archives of Toxicology

**Authors names:** Eiki Kimura^1^, Chieri Matsuyoshi^1^, Wataru Miyazaki^1^, Seico Benner^1^, Mayuko Hosokawa^2^, Kazuhito Yokoyama^2^, Masaki Kakeyama^1, 3^, and Chiharu Tohyama^1^

**Affiliation:** ^1^Laboratory of Environmental Health Sciences, Center for Disease Biology and Integrative Medicine, Graduate School of Medicine, The University of Tokyo, Tokyo, Japan; ^2^Department of Epidemiology and Environmental Health, Juntendo University Faculty of Medicine; ^3^Graduate School of Biomedical Science, Nagasaki University, Nagasaki, Japan

**E-mail address of the corresponding author:** Chiharu Tohyama, [mtohyama@mail.ecc.u-tokyo.ac.jp](mailto:mtohyama@mail.ecc.u-tokyo.ac.jp); Masaki Kakeyama, [kakeyama@nagasaki-u.ac.jp](mailto:kakeyama@nagasaki-u.ac.jp)


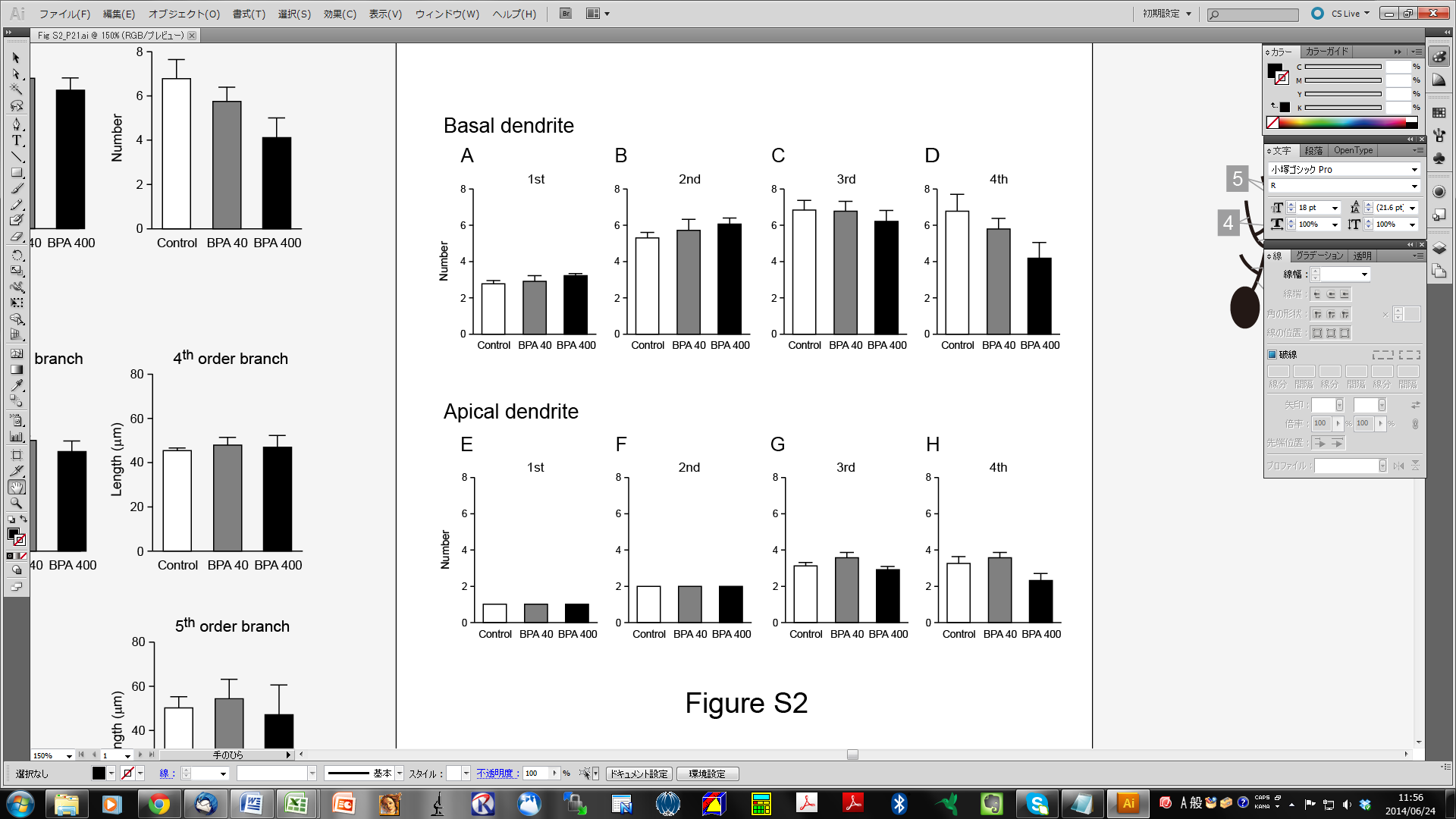


**Fig. S1** Branching numbers of basal and apical dendrites of hippocampal CA1 pyramidal neurons of 21-day-old GFP-Thy1-M mice prenatally exposed to BPA. First- to fourth-order branching of basal (A–D) and apical dendrites (E–H) on CA1 pyramidal neurons. Values are shown as means ± S.E.M for five mice per group.
